# Delivering Diagnostic Quality Video over Mobile Wireless Networks for Telemedicine

**DOI:** 10.1155/2009/406753

**Published:** 2009-05-04

**Authors:** Sira P. Rao, Nikil S. Jayant, Max E. Stachura, Elena Astapova, Anthony Pearson-Shaver

**Affiliations:** ^1^AEC Automotive, Texas Instruments Inc. Stafford, TX 77477, USA; ^2^School of Electrical and Computer Engineering, Georgia Institute of Technology, Atlanta, GA 30332, USA; ^3^Center for Telehealth, School of Medicine, Medical College of Georgia, Augusta, GA 30912, USA; ^4^Department of Pediatrics, School of Medicine, Medical College of Georgia, Augusta, GA 30912, USA

## Abstract

In real-time remote diagnosis of emergency medical events, mobility can be enabled by wireless video communications. However, clinical use of this potential advance will depend on definitive and compelling demonstrations of the reliability of diagnostic quality video. Because the medical domain has its own fidelity criteria, it is important to incorporate diagnostic video quality criteria into any video compression system design. To this end, we used flexible algorithms for region-of-interest (ROI) video compression and obtained feedback from medical experts to develop criteria for diagnostically lossless (DL) quality. The design of the system occurred in three steps-measurement of bit rate at which DL quality is achieved through evaluation of videos by medical experts, incorporation of that information into a flexible video encoder through the notion of encoder states, and an encoder state update option based on a built-in quality criterion. Medical experts then evaluated our system for the diagnostic quality of the video, allowing us to verify that it is possible to realize DL quality in the ROI at practical communication data transfer rates, enabling mobile medical assessment over bit-rate limited wireless channels. This work lays the scientific foundation for additional validation through prototyped technology, field testing, and clinical trials.

## 1. Introduction

Remote assessment of emergency medical events using telemedicine systems is likely to be a critical and pervasive component of future healthcare systems. Depending upon geography and economics, remote assessment is likely to occur over both wired and wireless networks, and thus over a broad spectrum of transmission bit rates ranging from very low to very high. Telemedicine systems that have the potential to include video when appropriate will provide specialists with significantly more information for assessment, diagnosis, and management than will systems without that capability. Bit-rate limitations often require that the compression ratio for video be larger than a certain minimum value. On the other hand, for medical video to be of diagnostic quality, the compression ratio must be smaller than a critical maximum value. In other words, it is essential for clinical acceptability that the compression process does not lead to misinterpretation because of a diagnostically relevant loss of detail or the introduction of visible artifacts. A number of compression schemes that attempt to ensure video of diagnostic quality have been reported in the telemedicine literature. The remainder of this section describes the state-of-the-art, with each new paragraph describing a unique literature domain.

**High-Bit Rate Local Area Network (LAN) Infrastructures for Telemedicine Systems**

Stamford et al. [1] address high-speed, high-quality video transmitted from a rural emergency department (ED) to a major medical center ED. The results document improved diagnosis and treatment, as well as improved confidence levels among doctors. Kofos et al. [2] studied telemedicine in pediatrics using a broadcast quality real-time audiovisual system and concluded that such a system may have dramatic implications for providing pediatric specialty and subspecialty care in underserved areas. Yoo [3] reported the design of an MPEG-2 video system running at 30 frames per second (fps) and requiring 1.5–6 megabits per second (Mbps) to deliver a spatial resolution of 640 × 480 pixels. At the peak bit rate, this corresponds to 0.434 bit per pixel (bpp). Qiao et al. [4] addressed the design of a critical care telemedicine system based on video over broadband networks. Wang et al. [5] described a web-based videoconferencing system that allows specialists at an academic medical center to evaluate stroke patients at a rural facility. Each of these systems describes a fixed patient at one location connected to a fixed physician at another location through a broadband connection. However, rural hospitals frequently have neither the equipment nor personnel infrastructure necessary to support high-bit rate broadband networks.

**Telemedicine Systems over Wireless Networks**

Because of the limitations due to fixed locations, a number of researchers have explored telemedicine systems over wireless networks in order to address mobility and the resulting infrastructure issues. Kugean et al. [6] discuss a telemedicine system design using a wireless local area network (WLAN). Encoded video is transmitted with (352 × 288) and (176 × 144) spatial resolution at 30 fps, requiring about 384 kilobits per second (kbps). This bit rate falls well within the WLAN capacity, but because bit rate is shared with other network users, the bit rate per user varies both with user number and time, with video quality often dropping to levels that create problems for physicians analyzing critical real-time patient features. Banitsas et al. [7] design faces similar issues. Chu and Ganz [8] describe a mobile teletrauma system based on wireless networks where video at 2–25 fps with 320 × 240 resolution is used. The network providers advertised a 153 kbps bit rate, but in fact the average available bit rate was only 50–60 kbps. Thus, in these types of systems, limited and varying bit rate availability are critical issues that are not compatible with clinical uses where the video quality must be maintained at a high and consistent level throughout the encounter.

**Nonmedical Region-of-Interest- (ROI-) Based Systems**

ROI processing is a potential alternative to address the constraints imposed by limited and varying bit rate systems. ROI processing refers the allocation of a greater bit rate per pixel to the ROI as compared to the background (BKGRND). Wong and Kwok [9] proposed a compression scheme for wireless channels where the channel bit rate is computed, and both ROI and BKGRND areas are treated with different and varying compression ratios. The BKGRND is dropped altogether when necessary. When the BKGRND is dropped completely, the best possible ROI quality under the circumstances is achieved, but physician experts then point out that the problem of context arises. Our discussions with collaborating Medical College of Georgia (MCG) physician experts revealed that while the ROI must be of *diagnostically* lossless quality for a clinical decision to be made using the video, simultaneous background context, or overview information is also required. They argued that background context, even of lower quality, is better than a black background. Further, if the consultant is providing care-related instructions to a clinician at the remote site who is actually handling the patient, the consultant will want to observe whether the correct manipulation is being made while simultaneously observing the patient response in the ROI. To summarize, DL quality in the ROI is necessary and is the most important issue, but is itself not a sufficient condition for the overall assessment of the patient. Chai et al. [10] proposed and implemented two ROI coding strategies. The maximum bit-transfer strategy assigns the highest compression level to the BKGRND, and the lowest possible compression level to the ROI that does not result in exceeding the overall available bit rate. This joint bit-allocation method allocates bits to the ROI and BKGRND based on the size, motion, and priority characteristics of each region.

**ROI-Based Telemedicine Systems**

In the medical domain, Gokturk et al. [11] proposed a compression scheme for 3D medical images where ROI is coded with lossless compression, and the BKGRND is coded in a “lossy” manner. The method was tested on computer tomography (CT) images of the human colon with the ROI being the diagnostically important colon wall. Gibson et al. [12] implemented ROI detection and bit allocation in a compression scheme for angiogram video sequences, with ROIs containing the coronary arteries of key diagnostic importance. Gokturk and Gibson techniques [11, 12] are proprietary approaches that do not relate to any particular video standard, and only pertain to medical data that have very low frame rates. They are not applicable to higher frame rate video which is the scope of our current work. The drawbacks of these ROI efforts [9, 10, 12, 13] lie in their arbitrary choices for ROI and BKGRND compression ratios. Diagnostic quality is not assured even within the ROI because of this arbitrary assignment.

**Telemedicine Systems Aimed at Achieving Diagnostic Level Quality**

A few studies [14–19] have specifically addressed the quality of medical images or video in telemedicine systems. Martini and Mazzotti [15] address the design of a video compression and transmission system for medical applications based on metrics such as peak-signal-to-noise-ratio (PSNR). PSNR, however, is a mathematical formula to measure the amount of compression in a video and has no definite relationship to diagnostic quality. Ashraf and Akbar [16] demonstrate diagnostically lossless (DL) medical images by using lossless coding within the ROI, but this cannot be extended to video because of bit-rate limitations. Gibson et al. [17] implement ROI compression on angiogram video sequences and verify that their results are of DL quality. However, this outcome is incidental and not actively pursued as part of the algorithm design. In summary, the authors are unaware of any work in literature within the context under consideration (wireless networks with bit rates in the 100 kbps—1 Mbps range, video at about 30 fps, spatial resolution at about 360 × 240 pixels) that specifically aims to develop algorithms to achieve diagnostic quality.

## 2. Elastic ROI Coding System for Diagnostically Lossless Quality

The goal of our elastic ROI coding algorithm is to enable mobile medical assessment. This is achieved by (1) incorporating physician expert feedback into the design of the video coding system in order to assess the bit rate at which DL video quality is achieved; (2) making the system operate with DL video quality at bit rates available in wireless networks using ROI-based bit allocation.

ROI definition (i.e., segmentation and tracking) is also an important problem in ROI system design that has been widely researched. Our framework is relatively simple. It involves a mounted camera in an emergency unit with standard lighting where the patient is located. Thus, for the purposes of our research, we focus on the more pressing issue of achieving DL video quality given a priori manual selection of the ROI by the physician experts.

### 2.1. Measurement of Bit Rate at which Diagnostically Lossless Quality is Achieved

Three levels of quality are postulated as follows.

- Mathematical losslessness (ML): the compression is lossless and therefore produces no loss of any digital information.
- Diagnostic losslessness (DL): there is lossy compression, but it does not compromise visual medical assessment in any way. In other words, the video quality is completely sufficient, according to physician experts, for making a diagnostic evaluation.
- Best effort (BE): this is the quality achieved with the highest possible compression in a video coding scheme (i.e., in a limited bandwidth environment, it is the poorest quality level).

Uncompressed color video requires 12 bpp, ML color video requires about 4–6 bpp, corresponding to lossless compression schemes in literature. BE quality typically requires about 0.1 bpp (with MPEG-2). Prior to our work, we found no literature that addresses the bit-rate requirement for DL. In other words, it is unknown how much lower the bit rate for DL level is in comparison to the ML level. We have published preliminary results of the proposed work [18, 19].

The procedure involves a set of videos compressed at a variety of bit rates and uniform spatial quality, that is, uniform compression. Physician experts blind to the degree of compression in the videos presented to them in random order identify maximum levels of compression that can be applied while retaining DL quality. The clinical experts complete an evaluation whose format is shown in Table 1, illustrated as an example specific to pediatric respiratory distress.

In Table 1, each sample number represents a video at a particular bit rate. The medical expert lists whichever features s/he can identify in the video. In the DL column, the four options relate to the quality of the video for clinical assessment of each feature set: 1-no, 2-maybe not, 3-likely, 4-yes. The lowest bit rate (TBR) at which the evaluation is 4 represents the threshold for DL *for the particular feature*. The sample partial table (Table 1) represents one video evaluated by one medical expert. The complete test set consists of several videos evaluated by several physicians. We also included a Comments column (not shown in Table 1) on the experts scoring sheets where they could give comprehensive qualitative feedback on the entire video content. The purpose of this column was to seek possible interactions among different features that might affect expert assessment of diagnostic quality. In our current algorithm development, we did not use the information from the Comments section because it was generally consistent with the quantitative feedback provided by the experts.

Our experts insisted on evaluating videos on a feature-by-feature basis, and not simply the video as a whole because, they explained, during in person evaluation they normally control the order of their inspection, often alternating between the inspection of an individual feature or finding and a general patient assessment. Further, they emphasized that in urgent situations, they would need the ability to control the order of inspection without the delays and potential for errors involved in communicating telephonic instructions to a remote and potentially clinically inexperienced camera holder. The design of the system was therefore developed with recognition of this requirement, and with the following logic. Each video was considered potentially unique in its information content. For example, a segment containing comprehensive frontal view of an infant possesses a wealth of information in terms of medical features, whereas a segment depicting the rear view of the infant head would be expected to contain more limited information. If two such video segments coded at several different bit rates are evaluated by clinical experts, evaluation of the overall video would still lead to limited information unless the clinician had the ability to examine closely the specific features visible in the over-all videos. This sequence is in fact how clinical evaluation is performed when the patient and the clinician are together in person.

If TBR_DLi represents the TBR required for DL for feature i averaged over all the physician experts, then the corresponding bpp value, denoted by bpp_DLi is

(1) $$\text{bpp}\_\text{DLi}=\frac{\text{TBR}\_\text{DLi}}{\text{FR}\_\text{RATE}\times \text{FR}\_\text{SIZE}},$$

where FR_RATE represents the frame rate (in fps) and FR_SIZE represents the spatial resolution of the videos (assuming one fixed value). To obtain one bpp value for DL encompassing all features, denoted by bpp_DL, the maximum bpp_DLi over all the features is chosen. This is a conservative approach that ensures that DL quality is achieved under all possible scenarios—visual information being encoded has lots of features, or few features.

In this work, we focus upon the problem of acute childhood respiratory distress as a representative example of an urgent need for remote consultation. With parental informed consent (MCG Human Assurance Committee no. 06-04-284), we collected 11 videos of pediatric patients in respiratory distress and used physician experts to complete evaluation templates (Table 1). Each video includes symptomatic features useful to physician experts for visual assessment. Each video has a spatial resolution of 360 × 240 pixels, a frame rate of 30 fps, and standard hospital Emergency Room lighting conditions.

The videos were encoded at uniform spatial quality using a standard MPEG-2 reference implementation at 3 bit rates—500 kbps, 1000 kbps, and 1500 kbps. Videos were then evaluated by MCG physician experts. Table 2 lists the evaluation of video Er08 by one of the experts. For simplicity of presentation here, the video sample names have been converted to the form VIDEONAME_TBR. Thus, Er08_500 corresponds to video Er08 encoded at 500 kbps. Er08_orig corresponds to the original uncompressed video. However, as stated previously, clinician evaluators were blind to the compression and videos were presented in random order.

Five videos were used to measure the bit rate at which DL quality is achieved. The remaining 6 videos were used for testing the efficacy of the proposed algorithm. Video Er08, although evaluated and displayed here, was left for testing purposes. After averaging over the 5 videos and all the experts, the required TBR values for DL for individual features were obtained and are tabulated in Table 3. Averaging over experts feedback is justified because they were closely clustered. We also observe the clustering of TBR values for DL for different medical features, irrespective of the patient race. Choosing the maximum TBR value results in DL over all features, and this value is 1077 kbps, that is, 0.42 bpp corresponding to the RR feature.

**Figure 1:**
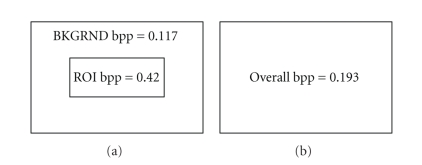
Illustration of bit per pixel density at 500 kbps and 360 × 240 pixels with (a) ROI compression: ROI bpp = 0.42; ROI bit rate = 0.42 × 360 × 240 × 0.25 × 30 = 270 kbps; BKGRND bit rate = 500 − 270 = 230 kbps; BKGRND bpp = 230000/(30 × 360 × 240 × 0.75) = 0.117 and (b) uniform compression: overall bpp = 500000/(30 × 360 × 240) = 0.193.

To put ROI compression in perspective with the above determined bpp value, Figure 1 illustrates the bit per pixel density at a TBR of 500 kbps with ROI compression and uniform compression of a frame of size 360 × 240 pixels. The ROI is assumed to be 25% of the total spatial size of the frame. Assume a frame rate of 30 frames per second. The numerical details are provided as illustrated in Figure 1.

Thus, with ROI compression, DL is achievable in the ROI even at 500 kbps, whereas it is not achievable with uniform compression. This is because the bit rate in the ROI at 500 kbps is 270 kbps, which is equivalent to an overall bit rate of 270 × 4 = 1080 kbps, which corresponds to the DL bit rate.

### 2.2. Encoder States to Map Quality Levels to ROI and BKGRND

The encoder must use the information on measurement of bit rate at which DL quality is achieved and map ML, DL, and BE quality levels to the ROI and BKGRND regions in the video. This is done using the notion of an encoder state which is defined by a pair of quality levels, one corresponding to the ROI and the other corresponding to the BKGRND. The complete state table for the encoder is shown in Table 4. In the table, the states are numbered from 1 to 6 according to decreasing priority, based on the basic premise that the ROI quality should be at least the same as or better than the BKGRND quality. For example, state 5 represents DL quality ROI and BE quality BKGRND. Also, some of the entries in the state table are marked N/A, that is, not applicable because they violate the basic premise that ROI quality must match or exceed BKGRND quality.

The encoder state of operation is determined through a bit-allocation algorithm between the ROI and BKGRND. The encoder state is determined or updated over every group of video frames (pictures), referred to as a GOP. Typically, there are 15 frames in a GOP. State determination is done at the GOP level in order to average over its constituent frames which typically have different levels of compression. For the video currently being encoded, the nominal number of bits required per GOP for DL in the ROI denoted by R_ROI_DL is

(2) R_ROI_DL=bpp_DL×ROI_SIZE×N_GOP,

where ROI_SIZE is the average size of the ROI for the current GOP and may be a window of standard shape and size around the feature(s). N_GOP denotes the number of frames in a GOP. Similar expressions hold for the nominal number of bits required for other quality levels for the ROI and BKGRND. For ML and BE quality levels, bpp_ML and bpp_BE values documented in literature are used.

**Figure 2:**
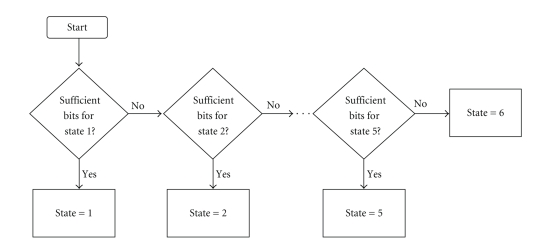
Flow diagram describing encoder state determination.

At the beginning of a new GOP, the encoder tries to occupy the highest priority state given the current TBR. If R denotes the target number of bits in a GOP (which is directly proportional to the TBR), the encoder checks if it can occupy state 1, governed by the condition: R > R_ROI_ML + R_BKGRND_ML. If so, it occupies state 1, otherwise it checks if it can occupy state 2, governed by the condition: R > R_ROI_ML + R_BKGRND_DL. This procedure continues until it occupies one of the six possible states, and is illustrated in Figure 2. 

**Figure 3:**
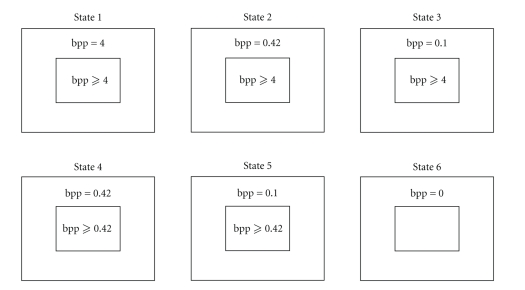
ROI and BKGRND bit per pixel density in states 1 through 6.

Figure 3 is analogous to Figure 1 and depicts the bit per pixel density in the ROI and BKGRND in each state. In each state, the BKGRND is allocated bits first, and then the remaining bits are allocated to the ROI. This is done to ensure that the ROI gets bits in excess of the nominal value allocated to the ROI in that state. For example, in state 5, where the ROI has DL quality and BKGRND has BE quality, the allocation is done as

(3) $$\begin{array}{c} \text{R}\_\text{BKGRND}=\text{R}\_\text{BKGRND}\_\text{BE},\\ \text{R}\_\text{ROI}=\text{R}-\text{R}\_\text{BKGRND}, \end{array}$$

where R_ROI and R_BKGRND denote the allocated number of bits in a GOP for the ROI and BKGRND, respectively. If the encoder determines that state 5 is the current GOP state, the BKGRND gets allocated R_BKGRND_BE bits, and the ROI gets allocated *at least* R_ROI_DL bits. In state 6, the TBR is low, so all the bits are allocated to the ROI, but we still do not achieve DL quality.

At a given TBR, the encoder occupies a particular state, which is the highest priority under the given conditions. When the TBR changes, as in a variable bit-rate (VBR) wireless channel, the encoder may transition to a new state at the beginning of a new GOP. Depending on the change in TBR, the encoder may move to a higher priority state or a lower priority state.

### 2.3. Encoder State Update

The encoder operation is based on bit allocations to ROI and BKGRND based on the nominal bpp_DL value obtained in Section 2.1 above. The encoder is said to have DL quality in the ROI if its current state corresponds to a DL quality for the ROI, that is, states 4 and 5. The bit allocations in these states are

(4) $$\begin{array}{ll} \text{STATE}\;\;4: & \;\;\text{R}\_\text{BKGRND}=\text{R}\_\text{BKGRND}\_\text{DL},\\ & \;\;\text{R}\_\text{ROI}=\text{R}-\text{R}\_\text{BKGRND},\\ \text{STATE}\;\;5: & \;\;\text{R}\_\text{BKGRND}=\text{R}\_\text{BKGRND}\_\text{BE},\\ & \;\;\text{R}\_\text{ROI}=\text{R}-\text{R}\_\text{BKGRND}. \end{array}$$

For a given value of R, R_ROI is higher in state 5 than in state 4 because R_BKGRND_DL is larger than R_BKGRND_BE. To account for any possible deviation from nominal DL quality behavior, more bits are allocated to the ROI whenever possible and deemed necessary. If the encoder state is 4, then ROI bits are increased by transitioning to state 5. In state 5, R_ROI is increased by reducing R_BKGRND to a lower value R_BKGRND_BE_MIN. The strategy used to determine when the above transition is necessary is based on the ROI PSNR value. This value is compared to the average PSNR obtained at the DL bit rate for the features in Section 2.1.

In states 4 and 5, whenever the ROI PSNR falls below this PSNR threshold, a state transition flag is set to 1. Then, in the following GOP, if the encoder state is determined (based on the approach illustrated in Figure 2) to be either 1, 2, 3, or 6, the flag is discarded because the wireless channel has determined a new quality level (ML or BE) for the ROI. If the current state is determined to be 4 or 5 and the previous state was 4, the new state is 5. If the previous state was 5 and the current state is determined to be 4, the new state is set to 5. If the previous state was 5 and the current state is determined to be 5, the new state is also 5 but R_BKGRND is lowered to R_BKGRND_BE_MIN. These rules are summarized in Table 5.

## 3. Evaluation of the Diagnostic Quality Encoding System

In this section of our work, we compress the 6 test videos (not used in Section 2.1) using the proposed elastic bit-allocation algorithms implemented within an MPEG-2 encoder. The bpp values for DL and BE were used to do ROI encoding of the test videos at three different TBR—500 kbps, 750 kbps, and 1000 kbps. These bit rates correspond to the wireless sweet spot, that is, representative bit rates in state-of-the-art wireless technologies. The ROI was specified by the medical experts depending on the video scene, and its size typically varied from 25–50% of the total spatial resolution. The evaluation template provided to the experts for the test videos is identical to that presented in Table 1, rendering our ROI coding process transparent to the expert.

Table 6 shows the completed evaluation template by a medical expert for video Er08. A comparison with Table 2 reveals improvement in performance at 500 kbps over the uniform compression case. This is true since the corresponding values in Table 7 are populated with 3 seconds and 4 seconds indicative of DL quality whereas the values in Table 2 are populated with 2 seconds indicative of non-DL quality.

**Figure 4:**
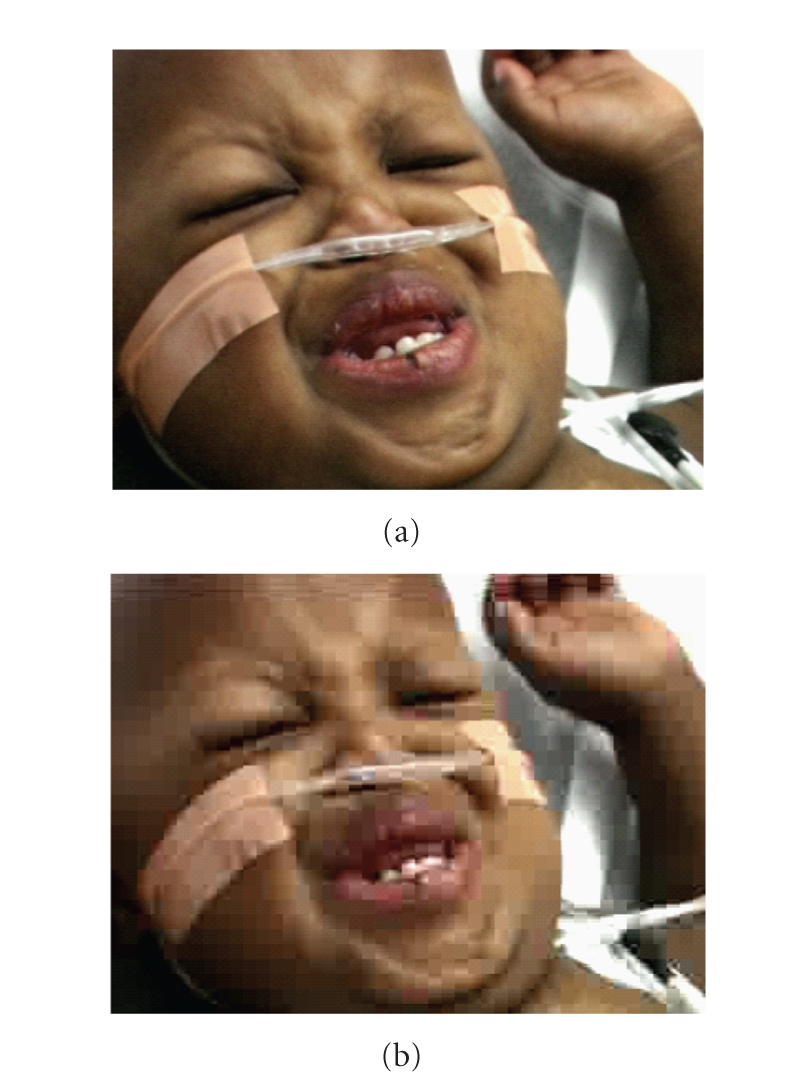
Video Er08. (a) Uncompressed video and (b) Uniform compression at 500 kbps.

**Figure 5:**
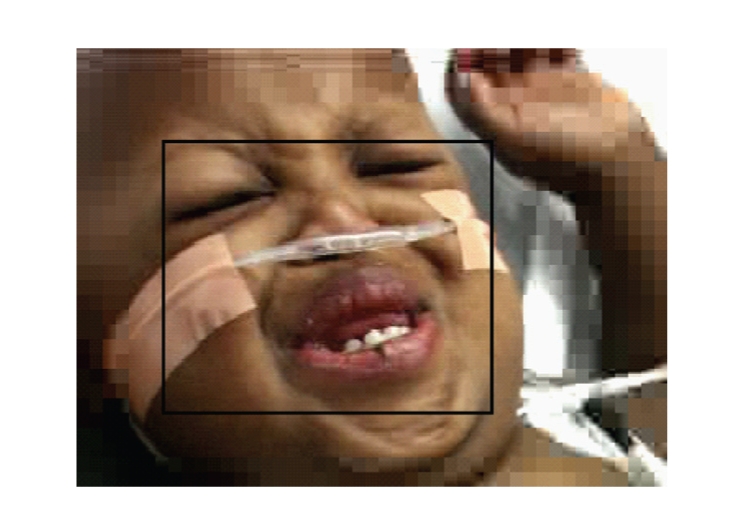
Representative ROI-encoded frame of video Er08 at 500 kbps with elastic bit allocation.

Figure 5 shows a frame representative of overall quality of video Er08 at 500 kbps with elastic ROI-based bit allocation. This figure may be compared to Figure 4 to compare with the same frame when uncompressed (Figure 4(a)) and when uniformly compressed at 500 kbps (Figure 4(b)). It may be observed that the BKGRND bit rate is lower in Figure 5 compared to Figure 4(b). However, visually, the artifacts are prominent to about the same degree in both cases. This is because artifacts do not get too much worse when the bit rate is small. However, the key is the achievement of a high level of quality in the ROI.

To gain an understanding of the general performance of our method, the above feedback was considered for all the test videos. These were then averaged to obtain a mean (*μ*) and standard deviation (*σ*) score for DL at 500 kbps, 750 kbps, and 1000 kbps. Table 7 shows these values. Since DL is expected within the ROI (unless the encoder is in state 6) irrespective of the particular medical feature, the features column does not appear in the table. At 500 kbps, 750 kbps, and 1000 kbps, *μ* DL values are 3.67, 3.78, and 3.79, respectively. The standard deviation values are indicative of the variability about the mean values. With uniform compression at 500 kbps, *μ* DL value is 2.29, with a standard deviation *σ* of 0.57. At 750 kbps, *μ* is 2.65, and *σ* is 0.59. At 1000 kbps, *μ* is 2.94, and *σ* is 0.64. Thus, with the proposed method, at 500 kbps the diagnostic quality of the video is 92%, compared to 57% with uniform compression.

## 4. Conclusion

Our work targets, and is relevant to, real-time remote diagnosis of emergency medical events using wirelessly transmitted videos of patients. This scenario is driven by the following: (a) the need for urgent evaluation of a distant patient that cannot be moved to the expert, or at least cannot be moved to the expert location in time to make a required critical decision, (b) the inadequacy of a still image to convey the clinical information necessary for decision-making, and (c) cost and infrastructure issues that preclude high-bit rate transmission of the captured video. We describe the design of an elastic video coding system, based on ROI, to achieve diagnostically lossless visual communications over wireless telemedicine networks. A novel algorithm is proposed that was developed using physician expert feedback to measure the bit rate at which DL quality is achieved. This bit rate is then incorporated into the encoder design which is based on operation in an optimal state at a particular TBR. The bit rate required for DL quality was determined to be 0.42 bpp for MPEG-2 video compression and 360 × 240 pixel resolution. It was validated by physician expert evaluation, and ROI encoded videos were deemed to be diagnostically lossless at 500 kbps, as compared to 1077 kbps for uniformly encoded videos. ROI encoding with our proposed algorithms results in DL video quality over the bit rate range corresponding to state-of-the-art wireless technologies.

## Figures and Tables

**Table 1:** Evaluation template to measure the bit rate at which DL quality is achieved.

Random	Feature sets	DL
video		1-no, 2-maybe not,
sample		3-likely, 4-yes
SAMPLE1	Activity	4
	Gasping	3
	Tachypnea	4
	⋯	⋯

**Table 2:** Evaluation of video Er08 compressed at four different compression levels (diagnostic level quality scale: 1-no, 2-maybe not, 3-likely, 4-yes).

Random	Feature sets	DL (1–4)
video sample		
Er08_500	WB	2
	RR	2
	T	2
	MS	2
	HB	2
	NF	2

Er08_1000	WB	3
	RR	3
	T	3
	MS	4
	HB	3
	NF	3

Er08_1500	WB	3
	RR	3
	T	3
	MS	4
	HB	4
	NF	4

Er08_orig	WB	4
	RR	4
	T	4
	MS	4
	HB	4
	NF	4

**Table 3:** Required bit rate for DL in medical features pertaining to pediatric respiratory distress.

Feature	TBR (kbps)
Activity (A)	750
Chest excursion (CE)	1000
Gasping (G)	1000
Level of activity (LA)	1000
Mild retraction (MR)	1000
Mental status (MS)	1000
Nasal flaring (NF)	750
**Respiratory rate (RR)**	**1077**
Retractions (R)	786
Tachypnea (T)	923
Work of breathing (WB)	750
Head bobbing (HB)	1000
Respiratory excursion (RE)	1000
Skin tone mottling (STM)	1000
SUPRA	500
INTER	500
SUB	750

**Table 4:** Encoder state table.

ROI quality ↓	BG quality →		
	ML	DL	BE
ML	1	2	3
DL	N/a	4	5
BE	N/a	N/a	6

**Table 5:** Encoder state update chart.

Previous state	State transition flag	Current state	New current state
		(as determined by Figure 1)	
4 or 5	0	1 or 2 or 3 or 4 or 5 or 6	Same as current state
4 or 5	1	1 or 2 or 3 or 6	Same as current state
4	1	4	5
4	1	5	5
5	1	4	5
5	1	5	5

**Table 6:** Evaluation of video Er08 when ROI coded (diagnostic level quality scale: 1-no, 2-maybe not, 3-likely, 4-yes).

Video_TBR	Features	Elastic (proposed)

		DL
Er08_500	WB	3
	RR	3
	T	3
	MS	4
	HB	4
	NF	3

Er08_750	WB	4
	RR	4
	T	4
	MS	4
	HB	4
	NF	4

Er08_1000	WB	4
	RR	4
	T	4
	MS	4
	HB	4
	NF	4

**Table 7:** Averaged results for DL at 500 kbps, 750 kbps, and 1000 kbps (diagnostic level quality scale: 1-no, 2-maybe not, 3-likely, 4-yes).

TBR	Elastic (proposed)	Uniform compression
	ROI DL (*μ*, *σ*)	DL (*μ*, *σ*)
500	3.67, 0.71	2.29, 0.51
750	3.78, 0.53	2.65, 0.59
1000	3.79, 0.44	2.94, 0.64
